# Retraction

**DOI:** 10.1111/jcmm.16259

**Published:** 2021-02-26

Retraction: MicroRNA‐181b negatively regulates the proliferation of human epidermal keratinocytes in psoriasis through targeting TLR4. Cheng Feng, Ming Bai, Nan‐Ze Yu, Xiao‐Jun Wang and Zeng Li. J Cell Mol Med. 21: 278‐285. (https://doi.org/10.1111/jcmm.12963).

The above article from *Journal of Cellular and Molecular Medicine*, published online on 19 September 2016 on Wiley Online Library (wileyonlinelibrary.com) in Volume 21, pp. 278‐285, has been retracted by agreement between the authors, the journal Editor‐in‐Chief and John Wiley & Sons Ltd. The retraction has been agreed after notification from the authors. The authors evaluated additional patients with psoriasis vulgaris after the publication of this paper. In the expanded study, it was found that the expression level of microRNA‐181b did not show a significant decrease in keratinocytes in patients with dandruff. It has been concluded that the result in the published paper is not valid due to the small number of cases studied in the initial study in 2013‐2015.

